# Breast milk and Group B streptococcal infection: Vector of transmission or vehicle for protection?

**DOI:** 10.1016/j.vaccine.2014.04.020

**Published:** 2014-05-30

**Authors:** Kirsty Le Doare, Beate Kampmann

**Affiliations:** a Imperial College London, Department of Paediatrics, St. Mary's Hospital, Praed Street, London, W2 1NY, UK; b Wellcome Trust Centre for Global Health Research, Norfolk Place, London, UK; c MRC Unit, Vaccinology Theme, Atlantic Road, Fajara, The Gambia

**Keywords:** Group-B streptococcus, Breast milk, Immunity, Antibody, Vaccination

## Abstract

Invasive Group-B streptococcal (GBS) disease is a leading cause of infant mortality and morbidity worldwide. GBS colonises the maternal rectum and vagina and transmission of bacteria from a colonized mother to her infant at birth is an important risk factor for GBS disease. GBS disease has also been associated with case reports of transmission via infected breast milk raising questions about mode of acquisition and transmission of this enteric pathogen and the development of neonatal disease. However, most breastfed infants remain unaffected by GBS in breast milk. Mechanisms associated with transmission of GBS in breast milk and potential factors that may protect the infant from transmission remain poorly understood. Understanding factors involved in protection or transmission of GBS infection via breast milk is important both for premature infants who are a high-risk group and for infants in the developing world where breastfeeding is the only sustainable infant feeding option. In this review we discuss the proposed mechanisms for GBS colonization in breast milk on one hand and its immune factors that may protect from transmission of GBS from mother to infant on the other. Innate and adaptive immune factors, including serotype-specific antibody and their significance in the prevention of infant disease are presented. We further report on the role of human oligosaccharides in protection from invasive GBS disease. Advances in our knowledge about breast milk and immunity in GBS disease are needed to fully appreciate what might mitigate transmission from mother to infant and protect neonates from this devastating disease and to contribute to the development of novel prevention strategies, including maternal immunization to prevent infant disease.

## Highlights

• GBS has been implicated in late onset-disease cases associated with breast milk.
• Proposed mechanisms include transfer of bacteria from the colonization of the infant to the milk ducts during suckling.
• Serotype-specific IgA antibody to GBS in breast milk may provide additional protection from invasive GBS disease.
• Vaccination of mothers in pregnancy may enable higher GBS-antibody concentrations to pass in breast milk.

## 1 Introduction

*Streptococcus agalactiae* (Lancefield Group B streptococcus; GBS) was first described as a cause of bovine mastitis by Nocard and Mollereau in 1887 [1]. Lancefield and Hare subsequently identified GBS in vaginal swabs in 1935 [2] and in 1938 Fry described three fatal cases in post-partum women [3]. Reports of neonatal disease from GBS were sporadic until the early 1960s when GBS became recognized as a leading cause of early neonatal sepsis in the USA [4]. By the 1970s it had become the dominant pathogen in the early neonatal period [5]. By the early 1980s GBS had become the most common cause of neonatal sepsis and meningitis in a number of developed countries [6–8]. In the past five years, late-onset (LO) GBS disease has been associated with case reports of transmission via infected breast milk [9] raising questions about mode of acquisition and transmission of this enteric pathogen and the development of neonatal disease.

Although GBS is not just a neonatal disease, the disease incidence and severity is highest during the first 90 days of life. Early onset (EO) GBS disease (disease presenting in the first six days of life) accounts for approximately 60–70% of all GBS disease. GBS serotypes Ia, Ib, II, III and V are responsible for most EO disease [10,11]. In contrast, serotype III predominates in LO disease, which may be acquired perinatally, nosocomially or from the community. [12]

In the USA EO disease rates have declined from 1.4 per 1000 live births in 1990 [13] to at 0.28 per 1000 live births in 2012 [14] mainly attributed to the implementation of universal screening for GBS rectovaginal colonization in pregnant women and intrapartum antibiotic prophylaxis. However, the incidence of LO disease has remained static at between 0.3 and 0.4 per 1000 births since 1990 [14]. This amounts to 28,100 cases and 1865 deaths annually in the USA [14]. Although the epidemiology of GBS in resource-rich countries is well documented, its contribution to the burden of neonatal infection in low/middle income countries has proved more difficult to assess. GBS has been reported as the predominant cause of neonatal sepsis in South Africa and Kenya [15–17] as well as an important cause of meningitis in Malawi and Kenya, but Asian studies have reported a much lower incidence [18–20]. A recent systematic review reported that the overall incidence of GBS in resource-poor settings ranged between 0 and 3.06 per 1000 live births [21].

GBS colonizes the rectum and vagina, and maternal colonization is a pre-requisite for EO disease and a risk factor for LO [22,23]. In resource-rich countries an estimated 20–30% of pregnant women are colonized with GBS [23,24], approximately 50% of their babies become colonized and 1% progress to develop invasive disease. EO disease may occur rapidly; signs of sepsis are evident at birth or within 12 h in over 90% of cases (98% within the first 12 h) [12]. Despite its rarity, LO disease, mostly presenting as meningitis, has devastating long term consequences in survivors with up to 50% suffering severe neurological sequelae [25].

It has been suggested that GBS initially colonizes the infant's oropharyngeal mucosa when contact with maternal vaginal secretions occur at the time of birth [26]. Butter and DeMoor demonstrated GBS in the nose and throat of infants at the same time as GBS was cultured from the mother's breast milk [27]. Fileron et al. reviewed cases of LOGBS disease associated with GBS in breast milk and found 48 LOGBS disease cases between January 1977 and March 2013 of which four had no other positive culture from mother or infant other than GBS-contaminated breast milk. [9].

Therefore, there appears to be a dichotomy between cases of LO disease through infected breast milk and the potential benefits of the components of breast milk which protect the majority of infants from invasive disease. The underlying mechanisms of GBS transmission or protection through breast milk, are not fully understood, but are important to elucidate, particularly in the context of premature infants who are a high risk group and for infants in the developing world where breastfeeding is the only sustainable infant feeding option. In this review we focus on the peculiarities of GBS that may aid transmission in breast milk and the role of immune parameters such as antibody in breast milk on the other hand that may help protect the breastfed infant from GBS disease.

## 2 Breast milk as vector of transmission of GBS

### 2.1 GBS in breast milk

Few studies have identified presence of GBS in breast milk, and methodological differences make comparisons difficult [28–32]. Low incidence is described in mothers of extremely preterm infants of 0.4% [31] and term infants of 0.82%. Higher incidence in raw milk ranged from 3.5% [30] to 10% [29] in donor breast milk. However, the concurrent incidence of GBS colonization in these mothers and the effect of intrapartum and postpartum antibiotic treatment were unknown.

The variety of delivery, treatment and storage methods of breast milk offers potential for GBS contamination. Human breast milk may contain 10^3^ to 10^9^ cfu/mL of GBS at any point, representing a reservoir of potential infection for the neonatal gut [33]. Breast milk directly from the mother (either through natural breast feeding or as expressed breast milk) is given raw and is rarely cultured in cases of neonatal infection. Expressed breast milk and bank milk may be frozen, which affects immune components and bank milk may also be pasteurized. Pasteurization is thought to eradicate important viral and bacterial infections [34] but also depletes milk of the majority of its cellular components and immunoglobulins [35] and may increase the bacterial growth rate [36].

Very recently, best practices on the use of breast milk in the context of prevention of GBS neonatal disease have been proposed, including the search for GBS in milk at the time of recurrent GBS neonatal disease and in cases of mastitis in mothers of high-risk preterm neonates admitted to neonatal intensive care units [37]. For these neonates, microbiological control of raw milk has also been proposed in the absence of mastitis [37]. However, the best strategy has yet to be developed as it does not appear that pasteurizing maternal milk changes the overall incidence of late onset GBS disease in preterm infants [38]. In a recent review article of cases of late onset GBS disease from breast milk, GBS was found in 0–2% of raw milk samples and 1.4% of pasteurized milk samples [9].

### 2.2 Proposed methods of acquisition

Two main mechanisms of acquisition have been proposed: following colonization of the neonatal oropharynx at the time of birth, mothers may develop colonization of the milk ducts through ascending infection from the neonate, due to the retrograde flow of milk associated with suckling. The infant is then reinfected as the concentration of bacteria increases in the breast milk [39]. This may occur with or without mastitis depending on additional factors such as milk stasis and bacterial load [40]. In most of the case reports of GBS disease associated with breast milk there is no sign of maternal mastitis, indicating silent maternal duct colonization [9]. However, recent studies in animal models and discovery of lactobacilli in breast milk after oral administration suggest that bacteria from the maternal digestive tract may also colonize the breast. [41] It has also been suggested that lactic acid bacteria may transfer from the mother's gut to breast milk and through the milk to the infant's digestive tract [42]. The epidemiological relationship between neonatal and maternal derived GBS isolates in breast milk has been confirmed by polymerase chain reaction (PCR) [43]. However, it is not clear whether the LO disease relates to infected breast milk or is a result of gut translocation from an already colonized infant.

GBS may infect the submucosa of the gastrointestinal tract either through a defect in the epithelial cell layer, or by concomitant infectious agents [33]. As neonatal gastric acid secretion is reduced, more bacteria may reach the intestinal mucosa. This is supported by findings that preterm infants fed with contaminated maternal milk via nasogastric tube have developed GBS disease [44].

## 3 Breast milk as vehicle of protection from GBS infection

### 3.1 Innate and adaptive immune properties of breast milk

Breast milk is the main source of non-pathogenic bacteria to the infant gastrointestinal tract. Intestinal bacteria are one of the most important stimuli for the development of mucosa-associated lymphoid tissue (MALT) in the neonatal small intestine [45] and produce organic acids that prevent growth of enteric pathogens. Additionally, breast milk and colostrum contain many components with antimicrobial and immunomodulatory properties that are believed to impair translocation of infectious pathogens [46]. Some of these substances compensate directly for deficiencies in the neonatal immune system and enhance survival of defense agents, including secretory IgA (SIgA), lactoferrin, lysozyme, IFN-γ; some adapt the gastrointestinal tract to extrauterine life, i.e. epidermal growth factor [47]; some prevent inflammation or enhance specific-antibody production, such as PAF-acetylhydrolase, antioxidants, interleukins 1, 6, 8, and 10, transforming growth factor (TGF), secretory leukocyte protease inhibitors (SLPI), and defensin 1 [46]. Breast milk also contains substantial amounts of intracellular adhesion molecule 1 and vascular adhesion molecule 1; low quantities of soluble S-selectin, l-selectin and CD14, which may mediate differentiation and growth of B cells [46]. Natural autoantibodies, thought to be important in the selection of the pre-immune B cell repertoire and in the development of immune tolerance, are also detected in colostrum and in breast milk [48]. Recently, the beneficial effects of human oligosaccharides in prevention of neonatal diarrhoeal and respiratory tract infections have been highlighted [49,50].

Human breast milk is known to contain factors that can modulate toll-like receptor (TLR) signaling, including soluble TLR2, which can competitively inhibit signaling through membrane TLR2 [51], as well as a protein that inhibits TLR2-mediated and activates TLR4-mediated transcriptional responses in human intestinal epithelial and mononuclear cells by an as-yet-unknown mechanism [52]. It has been speculated that reduced TLR2 responsiveness at birth may facilitate the normal establishment of beneficial Gram-positive bifidobacteria intestinal flora. Lipids present in human milk have been shown to inactivate GBS in vitro, providing additional benefit to protect from invasive infection at the mucosal surfaces [53].

## 4 Antibody in breast milk

Neonates have low levels of SIgA and SIgM [54] thus protection from invasive pathogens at the mucosal surface relies on antibodies in breast milk. As antibody in breast milk is produced following antigenic stimulation of the maternal MALT and bronchial tree (bronchomammary pathway) [55], these antibodies are targeted to many infectious agents encountered by the mother both prior to birth and during the breastfeeding period.

It is currently hypothesized that SIgA represents the crucial primary protective component of breast milk [56,57]. SIgA protects against mucosal pathogens by immobilizing these, preventing their adherence to epithelial surfaces, or by neutralizing toxins or virulence factors. SIgA concentration is far higher in colostrum (12 mg/ml) than in that found in mature milk (1 mg/ml). A breastfed infant may ingest around 0.5–1.0 g of SIgA per day [40].

## 5 The role of SIgA in breast milk

SIgA production is enhanced by Interleukin-6 (IL-6) whilst the production of secretory components is enhanced by TNF-α and TGF-β causes class switching towards B cells producing IgA [47], all of which are present in breast milk.

SIgA antibodies present in breast milk are specific for numerous enteric and respiratory pathogens. In studies from resource-poor countries, breast milk-mediated protection against infections with *Vibrio cholerae*, *Campylobacter*, *Shigella*, *Giardia lamblia* and respiratory tract infections was significantly related to the content of SIgA antibody in breast milk against these pathogens [58–60]. This could support the hypothesis that similar protection could be obtained from SIgA antibody in breast milk to GBS in a highly breastfed population. However, maternal SIgA does not appear to enter the neonatal circulation, [61] except in preterm infants, where ingestion of milk rich in IgA to respiratory syncytial virus (RSV) resulted in increased serum IgA levels during the perinatal period [62], so its effectiveness is limited to the mucosal surface.

SIgA is more resistant to proteolysis than other immunoglobulins and is therefore able to function in the gastrointestinal tract [46]. This could account for the finding that the faeces of breast fed infants contains IgA by the second day of life, compared to 30% of formula-fed infants, where IgA is only found in faeces by one month of age [63].

Breast milk contains SIgA antibodies against bacterial-adhesion-site-like pili [46,64]. SIgA antibody in milk blocks adherence of *S. pneumoniae* and *Haemophilus influenza* to human retropharyngeal cells [64] and casein in vitro [65]. The neutralizing capacity of milk anti-poliovirus antibodies has also been reported [66,67].

The effect of third trimester maternal immunization with a single dose of licensed quadrivalent meningococcal vaccine on the potential protection of infants, including by breast milk demonstrated elevated *N. meningitidis-*specific IgA antibodies in breast milk up to six months post partum in vaccinated infants [68]. Similarly, in mothers who received pneumococcal polysaccharide vaccine (PSV) during the third trimester, the geometric mean concentration of IgA in breast milk was significantly higher two months postpartum than in women who received conjugate *H*. *influenzae* vaccine in the third trimester and remained higher at seven months post partum. [69]

## 6 Group B Streptococcal antibody in breast milk

As described above, high levels of breast milk SIgA could offer protection to neonates via interference of antibody with the carbohydrate-mediated attachment of GBS to nasopharyngeal epithelial cells. Through this mechanism, colonizing organism load may be reduced with a consequent reduction in morbidity and mortality caused by GBS in the neonatal period [70].

In transition milk, low or moderate IgA antibodies to CPS type III GBS, were detected in approximately 63% of a cohort of 70 Swedish women [71]. In a study of IgG antibody concentration in transition milk in 46 women from the USA, Weisman and Dobson [70] found concentrations of IgG to types Ia, II or III which were approximately 10% of those in maternal serum. Edwards et al. measured IgG and IgA in breast milk to type III GBS in 18 women with high and low antibody titers and found measurable levels of antibody in both groups up to 2 months post-delivery [72]. Detectable levels of CPS serotype III antibody in breast milk in women correlated with concurrently high levels in their serum. Whilst no studies demonstrate a correlation between GBS-antibody levels in breast milk and infant colonization, Berardi et al. report that GBS-positive breast milk is associated with heavy infant colonization [73].

To determine the effect of maternal immunization with GBS CPS-II and CPS-III antibody on postnatal protection from disease a rodent model has been used, where increased survival in pups exposed postnatally to breast milk with high titers of antibody compared to low titers was shown, supporting the beneficial added effect of breast milk antibody following vaccination [74,75].

## 7 Human oligosaccharides

Oligosaccharides prevent cell adherence for *S. pneumoniae* [76] and *Escherichia coli* (*E. coli*) [77]. Additionally, *E. coli* and *Campylobacter jejuni* toxin can be neutralized by oligosaccharides [49,78] and milk glycoconjugates prevent cell adherence of *Vibrio cholera* and *E. coli* [79,80]. Taken together, these studies suggest that the transfer of human milk oligosaccharides delivers real protection to infants against many bacterial and viral infections.

GBS type Ib and II polysaccharides are of interest as they are virtually identical to certain oligosaccharides present in human milk [75,81,82] which raises the possibility of cross-reactivity with other human glycoconjugates [83]. The results from murine models suggest that these oligosaccharides may act as receptor analogues that anchor the bacteria in the mucosal layer and prevent cell adhesion in the epithelial layer, thus preventing invasive disease.

## 8 Conclusion

Most neonatal infections occur via mucosal membranes in the respiratory, gastrointestinal, and urinary tracts, yet there is only limited protection at these vast mucosal surfaces during the neonatal period. Breast milk provides considerable amounts of specific SIgA antibodies that are produced as a result of microbial and food antigens the mother has previously encountered. Such SIgA antibodies from breast milk provide protection to the neonate at the mucosal surface. Breast milk additionally contains high concentrations of non-specific protective molecules, such as lactoferrin that has bactericidal, viricidal, and fungicidal properties. Milk oligosaccharides might block adherence of microorganism at the mucosal surface by functioning as receptor analogues.

There is increasing data from recent publications that enhanced protection against diarrhea, respiratory tract infections, otitis media and *H*. *influenzae* infections, as well as wheezing illness may persist for years after breastfeeding. However, the role of breast milk antibody in protection from neonatal GBS disease remains poorly understood. Current research is evaluating transport, persistence and function of GBS antibodies and other immune-constituents in breast milk. These studies aim to identify protective factors involved in the passive transfer of immune components in breast milk and associated protection from colonization and infant disease. Additionally, research correlating neonatal colonization with antibody levels in breast milk would provide insight into possibly protective factors from disease.

## Conflict of interest

None declared.

## Contributors

KLD developed the research idea, undertook the literature review and prepared the first draft of the manuscript. BK developed the research idea and substantially contributed to the drafting and revision of the manuscript.

## Funding

KLD is funded by a Wellcome Trust/Imperial Global Health Fellowship and the Royal College of Physicians Thomas Watt Eden Fellowship. BK is funded by the MRC and the NIHR.
